# Insects are a viable protein source for human consumption: from insect protein digestion to postprandial muscle protein synthesis in vivo in humans: a double-blind randomized trial

**DOI:** 10.1093/ajcn/nqab115

**Published:** 2021-05-21

**Authors:** Wesley J H Hermans, Joan M Senden, Tyler A Churchward-Venne, Kevin J M Paulussen, Cas J Fuchs, Joey S J Smeets, Joop J A van Loon, Lex B Verdijk, Luc J C van Loon

**Affiliations:** Department of Human Biology, NUTRIM School of Nutrition and Translational Research in Metabolism, Maastricht University Medical Centre+, Maastricht, The Netherlands; Department of Human Biology, NUTRIM School of Nutrition and Translational Research in Metabolism, Maastricht University Medical Centre+, Maastricht, The Netherlands; Department of Human Biology, NUTRIM School of Nutrition and Translational Research in Metabolism, Maastricht University Medical Centre+, Maastricht, The Netherlands; Department of Human Biology, NUTRIM School of Nutrition and Translational Research in Metabolism, Maastricht University Medical Centre+, Maastricht, The Netherlands; Department of Human Biology, NUTRIM School of Nutrition and Translational Research in Metabolism, Maastricht University Medical Centre+, Maastricht, The Netherlands; Department of Human Biology, NUTRIM School of Nutrition and Translational Research in Metabolism, Maastricht University Medical Centre+, Maastricht, The Netherlands; Laboratory of Entomology, Plant Sciences Group, Wageningen University, Wageningen, The Netherlands; Department of Human Biology, NUTRIM School of Nutrition and Translational Research in Metabolism, Maastricht University Medical Centre+, Maastricht, The Netherlands; Department of Human Biology, NUTRIM School of Nutrition and Translational Research in Metabolism, Maastricht University Medical Centre+, Maastricht, The Netherlands

**Keywords:** milk protein, FSR, stable isotopes, protein metabolism, intrinsically labeled protein

## Abstract

**Background:**

Insects have recently been identified as a more sustainable protein-dense food source and may represent a viable alternative to conventional animal-derived proteins.

**Objectives:**

We aimed to compare the impacts of ingesting lesser mealworm– and milk-derived protein on protein digestion and amino acid absorption kinetics, postprandial skeletal muscle protein synthesis rates, and the incorporation of dietary protein–derived amino acids into de novo muscle protein at rest and during recovery from exercise in vivo in humans.

**Methods:**

In this double-blind randomized controlled trial, 24 healthy, young men ingested 30 g specifically produced, intrinsically l-[1-^13^C]-phenylalanine and l-[1-^13^C]-leucine labeled lesser mealworm– or milk-derived protein after a unilateral bout of resistance-type exercise. Primed continuous l-[ring-^2^H_5_]-phenylalanine, l-[ring-3,5-^2^H_2_]-tyrosine, and l-[1-^13^C]-leucine infusions were applied, with frequent collection of blood and muscle tissue samples.

**Results:**

A total of 73% ± 7% and 77% ± 7% of the lesser mealworm and milk protein–derived phenylalanine was released into the circulation during the 5 h postprandial period, respectively, with no significant differences between groups (*P* < 0.05). Muscle protein synthesis rates increased after both lesser mealworm and milk protein concentrate ingestion from 0.025 ± 0.008%/h to 0.045 ± 0.017%/h and 0.028 ± 0.010%/h to 0.056 ± 0.012%/h at rest and from 0.025 ± 0.012%/h to 0.059 ± 0.015%/h and 0.026 ± 0.009%/h to 0.073 ± 0.020%/h after exercise, respectively (all *P* < 0.05), with no differences between groups (both *P* > 0.05). Incorporation of mealworm and milk protein-derived l-[1-^13^C]-phenylalanine into de novo muscle protein was greater after exercise than at rest (*P* < 0.05), with no differences between groups (*P* > 0.05).

**Conclusions:**

Ingestion of a meal-like amount of lesser mealworm–derived protein is followed by rapid protein digestion and amino acid absorption and increases muscle protein synthesis rates both at rest and during recovery from exercise. The postprandial protein handling of lesser mealworm does not differ from ingesting an equivalent amount of milk protein concentrate in vivo in humans.

This trial was registered at www.trialregister.nl as NL6897.

See corresponding editorial on page 833.

## Introduction

It has been well established that ingestion of protein increases muscle protein synthesis rates (1). The anabolic properties of various protein sources (2–5) are largely defined by their protein digestion and amino acid absorption kinetics (3, 6–8) and amino acid composition (6, 9, 10). Physical activity and exercise performed before protein ingestion can further augment the muscle protein synthetic response to feeding (5, 11–13).

Dietary protein sources can be broadly classified as either animal- or plant-based proteins. Animal-based proteins are generally considered to have stronger anabolic properties than isolated plant-derived proteins (14). This has been attributed to the fact that isolated plant-derived proteins often show an incomplete amino acid profile with low amounts of leucine and/or insufficient lysine, histidine, or methionine (14). The few studies that have compared postprandial muscle protein synthesis rates after ingestion of isolated plant- and animal-derived proteins have reported a lesser increase in muscle protein synthesis rates after ingestion of wheat (15) and soy (10, 12, 16, 17) protein than after dairy protein. However, with regards to sustainability, the production of sufficient amounts of conventional animal-based proteins to meet future global food demands represents a challenge. Consequently, there is a growing interest in the definition of other, (more) sustainable protein sources. Edible insects have gained much interest as an alternative source of dietary protein that may be produced on a more viable and sustainable commercial scale and, as such, may contribute to ensuring global food security (18, 19).

Insects have a high protein content and their amino acid composition indicates that insects may provide a high-quality protein source that can stimulate postprandial muscle protein accretion similarly to high-quality animal-based protein sources (18, 20, 21). Lesser mealworm (*Alphitobius diaperinus* Panzer; Coleoptera: Tenebrionidae)–derived protein has an amino acid composition that closely resembles protein sources such as milk or meat (18, 22). So far, to our knowledge there are no data available on lesser mealworm protein digestion and amino acid absorption kinetics or on the postprandial anabolic response to lesser mealworm protein ingestion. We hypothesize that lesser mealworm protein is properly digested and absorbed, and has the capacity to increase muscle protein synthesis rates in vivo in humans both at rest and during recovery from exercise.

To test our hypotheses, we first fed lesser mealworm larvae with free l-[1-^13^C]-phenylalanine and l-[1-^13^C]-leucine to produce intrinsically l-[1-^13^C]-phenylalanine and l-[1-^13^C]-leucine labeled lesser mealworm protein. Combining the ingestion of intrinsically l-[1-^13^C]-phenylalanine and l-[1-^13^C]-leucine labeled lesser mealworm or milk protein concentrate (23–25) with the intravenous infusion of l-[ring-^2^H_5_]-phenylalanine and l-[1-^13^C]-leucine allowed us to quantify protein digestion and amino acid absorption kinetics, postprandial muscle protein synthesis rates, as well as the incorporation of dietary protein–derived amino acids into de novo muscle protein in vivo in humans (26).

## Methods

### Production of intrinsically labeled milk- and lesser mealworm–derived protein

Previously, we (25, 27, 28) and others (29–31) have produced different types of intrinsically labeled protein. Intrinsically labeled milk protein was produced by infusing lactating dairy cows with large quantities of l-[1-^13^C]-leucine and l-[1-^13^C]-phenylalanine. Throughout the stable isotope infusion milk was subsequently collected, processed, and fractioned as described previously (25, 32, 33), providing intrinsically l-[1-^13^C]-phenylalanine [38.3 mole percentage excess (MPE)] and l-[1-^13^C]-leucine (10.8 MPE) labeled milk protein.

For the current study, we applied the same principle to produce intrinsically l-[1-^13^C]-phenylalanine and l-[1-^13^C]-leucine labeled lesser mealworm. A 2.4-kg batch of 21-d-old lesser mealworm larvae (*A. diaperinus*; Protifarm) were fed 2.4 kg chicken feed (mainly wheat, barley, sorghum, carrot pulp, and apple core) mixed with 16 g l-[1-^13^C]-leucine and 103 g l-[1-^13^C]-phenylalanine. After 7 d, when the larvae had significantly increased in size, a sample was taken for microbiological testing to confirm that the larvae met all chemical and bacteriological specifications for human consumption (Eurofins KBBL). The larvae were sieved from the feed substrate, deprived of food for 3 h to empty their gut content, and then sieved again. Subsequently, the larvae were blanched and freeze-dried for 48 h to provide ∼800 g dry lesser mealworm. The freeze-dried lesser mealworm larvae were ground into a fine powder in an experimental kitchen with food-grade status (Human Nutrition Research Unit, Wageningen University). Subsequently, nitrogen content (Dumas method; 34) and amino acid composition (ultra-performance LC–MS; ACQUITY UPLC H-Class with QDa; Waters) of the freeze-dried lesser mealworm powder were measured. The l-[1-^13^C]-leucine, l-[1-^13^C]-phenylalanine, and l-[1-^13^C]-tyrosine enrichments of the freeze-dried lesser mealworm powder were measured in triplicate by GC-MS (Agilent 7890A GC/5975C; MSD) after overnight hydrolysis (9.3, 50.8, and 33.4 MPE, respectively).

### Subjects

Twenty-four healthy, young men [mean ± SD age: 23 ± 3 y; BMI (in kg/m^2^): 23.1 ± 2.7] were selected to participate in this study. **Table 1** details the characteristics of the subjects. The trial was conducted between February 2018 and May 2019 at Maastricht University Medical Centre+, in Maastricht, Netherlands. All subjects were informed of the nature and possible risks of the experimental procedures, before providing written informed consent. This study was approved by the Medical Ethical Committee of the Maastricht University Medical Centre+ and performed in conformance with the principles outlined in the Declaration of Helsinki for use of human subjects and tissue.

**TABLE 1:** Subjects’ characteristics^1^

	WORM	MILK	*P* value
Age, y	24 ± 3	22 ± 3	0.156
Body mass, kg	77.6 ± 11.1	72.0 ± 8.6	0.179
Lean body mass, kg	60.4 ± 7.0	56.6 ± 5.6	0.395
Appendicular lean mass, kg	28.2 ± 3.3	26.4 ± 3.4	0.414
Body fat, %	20.1 ± 5.4	17.9 ± 3.9	0.693
Height, m	1.70 ± 0.07	1.80 ± 0.10	0.712
BMI, kg/m^2^	24.2 ± 2.8	22.1 ± 2.2	0.061
Systolic blood pressure, mm Hg	123 ± 10	122 ± 7	0.827
Diastolic blood pressure, mm Hg	70 ± 8	69 ± 4	0.743
Resting heart rate, bpm	61 ± 8	58 ± 7	0.397
1-RM Leg press, kg	143 ± 29	139 ± 28	0.694
1-RM Leg extension, kg	62 ± 9	60 ± 11	0.410

^1^Values are means ± SDs. *n* = 12/group; data for lean body mass, appendicular lean mass, and body fat are displayed for *n* = 10 in the WORM group. Data were analyzed using an independent-samples *t* test. No differences were detected between groups. MILK, 30 g milk protein concentrate; WORM, 30 g lesser mealworm–derived protein; 1-RM, 1-repetition maximum.

### Pretesting

Volunteers aged between 18 and 35 y with a BMI between 18.5 and 30.0 kg/m^2^ underwent medical screening to assess body mass, height, blood pressure, and body composition (via DXA; Discovery A, Hologic). The participants were deemed healthy and eligible to participate based upon their response to a medical questionnaire and screening results. Participants were excluded if they were smoking, using medication that affected protein metabolism, intolerant to the investigated proteins, performing structural resistance training, or exercising >5 times/wk. After initial screening participants were familiarized with the exercise testing protocol and exercise equipment. Participants underwent estimates of unilateral 1 repetition maximum (1-RM) strength on the supine leg press and seated leg extension (Technogym BV). After a 1-legged warm-up on a cycle ergometer (Lode BV) at 75 W for 5 min with the assigned exercise leg, 10 submaximal repetitions were performed on the exercise machine. Next, the subject performed single repetitions with incremental weights for every repetition until failure. Between every attempt, a rest period of ≥2 min was taken and the 1-RM was typically reached between 5 and 7 attempts.

### Study design

In this double-blind, randomized, parallel-group trial, subjects were randomly assigned to consume either 30 g intrinsically l-[1-^13^C]-phenylalanine and l-[1-^13^C]-leucine labeled milk protein (MILK; *n* = 12) or 30 g intrinsically l-[1-^13^C]-phenylalanine and l-[1-^13^C]-leucine labeled lesser mealworm–derived protein (WORM; *n* = 12). Randomization was performed using a computerized random-number generator. An independent person was responsible for blinding and drink preparation. Within each treatment group the exercise leg was randomly assigned. In each group 6 subjects exercised with their nondominant and 6 with their dominant leg. All presented muscle data for the WORM group are based on *n* = 11, because of missing muscle biopsies in 1 of the participants. Data derived from the DXA scan were only available for *n* = 10 in the WORM group, because of logistical issues.

### Diet and physical activity

All subjects refrained from any sort of strenuous physical activity or exercise for 3 d before the trial and kept their diet as consistent as possible for 2 d before the experiment. Mean ± SD habitual protein intake was 1.5 ± 0.6 g protein · kg body mass^−1^ · d^−1^. On the evening before the experimental trial at 20:00, all subjects consumed the same standardized meal (1.71 MJ/405 kcal) providing 22 energy percent (En%) protein, 53 En% carbohydrate, and 26 En% fat.

### Intrinsically labeled protein

Subjects ingested 30 g intrinsically l-[1-^13^C]-phenylalanine and l-[1-^13^C]-leucine labeled lesser mealworm– or milk-derived protein. To allow ingestion of 30 g protein, subjects ingested either 64 g powdered lesser mealworm or 40 g dried milk protein concentrate dissolved into 300 mL water. The nitrogen content of the ingested lesser mealworm and milk protein concentrate was 10% and 12%, respectively, and conversion factors of 4.76 and 6.25 were used to calculate the protein contents of the dried lesser mealworm (47%) and milk protein concentrate (75%), respectively (35). The dried lesser mealworm provided 312 kcal, with 46En%, 6En%, and 46 En% provided by protein, carbohydrate, and fat, respectively (Kreca). Milk protein concentrate provided 142 kcal, with 91En%, 6En%, and 5 En% provided by protein, carbohydrate, and fat, respectively (Friesland Campina). **Table 2** shows the amino acid composition of the lesser mealworm– and milk-derived protein. Drinks were prepared and provided to the participants in a nontransparent shaker bottle by an independent person. After ingestion, bottles were rinsed with 50 mL water and the remaining fluid was ingested to ensure that all protein was consumed.

**TABLE 2:** Milk- and lesser mealworm–derived protein characteristics^1^

Component	WORM	MILK
Alanine, g	2.3	1.0
Arginine, g	1.6	0.9
Aspartic acid, g	2.1	1.7
Glutamic acid, g	3.6	6.1
Glycine, g	1.6	0.5
Histidine, g	0.9	0.7
Isoleucine, g	0.9	1.0
Leucine, g	2.5	2.8
Lysine, g	1.8	2.2
Methionine, g	0.4	0.8
Phenylalanine, g	1.2	1.2
Proline, g	1.8	3.4
Serine, g	1.4	1.3
Threonine, g	1.2	1.1
Tyrosine, g	2.4	1.4
Valine, g	1.3	1.2
EAAs, g	10.2	11.0
NEAAs, g	16.9	16.3
TAAs, g	27.2	27.2
l-[1-^13^C]-leucine, MPE	9.3	10.9
l-[1-^13^C]-phenylalanine, MPE	50.8	38.3
l-[1-^13^C]-tyrosine, MPE	33.4	8.6

^1^Asparagine, cysteine, glutamine, and tryptophan were not measured. EAA, essential amino acid; MILK, 30 g milk protein concentrate; MPE, mole percentage excess; NEAA, nonessential amino acid; TAA, total amino acid; WORM, 30 g lesser mealworm–derived protein.

### Study protocol

At 07:30, after an overnight fast, subjects arrived at the laboratory by car or public transport. A catheter was inserted into an antecubital vein for stable isotope–labeled amino acid infusion. A second catheter was inserted into a dorsal hand vein of the contralateral arm and placed in a hot box (60°C) for arterialized blood sampling (36). After taking a baseline blood sample, the plasma amino acid pools were primed with a single dose of l-[ring-^2^H_5_]-phenylalanine (2.0 µmol/kg), l-[ring-3,5-^2^H_2_]-tyrosine (0.613 µmol/kg), and l-[1-^13^C]-leucine (3.99 µmol/kg), after which a continuous intravenous l-[ring-^2^H_5_]-phenylalanine (0.05 µmol · kg^−1^ · min^−1^), l-[ring-3,5-^2^H_2_]-tyrosine (0.015 µmol · kg^−1^ · min^−1^), and l-[1-^13^C]-leucine (0.1 µmol · kg^−1^ · min^−1^) infusion was initiated (*t* = −180 min). Subsequently, the subjects rested in a supine position for 120 min during which 2 additional arterialized blood samples were drawn (*t = −*120 and −60 min).

At *t = −*50 min, the exercise session was started. The exercise session consisted of 1-legged cycling at 75 W for 5 min followed by 5 sets of leg press and leg extension. For the first set, the workload was set at 50% of the predetermined 1-RM for 10 repetitions. The following 3 sets were executed at 80% 1-RM for 8–10 repetitions, with the fifth set being performed until volitional failure. The subjects rested for 2 min between all sets and exercises and were verbally encouraged to complete the protocol.

Immediately after the exercise, another arterialized blood sample was collected together with a muscle biopsy sample from the *M. vastus lateralis* of both the exercised (EXERCISE) as well as the rested (REST) leg. Immediately thereafter subjects consumed 30 g intrinsically l-[1-^13^C]-phenylalanine and l-[1-^13^C]-leucine labeled lesser mealworm– or milk-derived protein (*t =* 0 min). Arterialized blood samples were collected at *t* = 20, 40, 60, 90, 120, 150, 180, 240, and 300 min. Additional muscle biopsy samples were obtained at *t* = 120 and 300 min from both legs to determine mixed muscle protein synthesis rates both at rest and after exercise.

Blood samples were collected in EDTA-containing tubes and centrifuged at 1000 × *g* at 4°C for 10 min. Aliquots of plasma were frozen in liquid nitrogen and stored at −80°C until further analysis. Muscle biopsy samples were taken from the middle region of the *M. vastus lateralis*, 15 cm above the patella and 3 cm below entry through the fascia, using a modified Bergström needle (37). Muscle samples were dissected carefully, freed from any visible fat, immediately frozen in liquid nitrogen, and stored at −80°C until further analysis.

### Plasma and muscle tissue analyses

The **Supplemental Methods** present details of analyses related to the determination of plasma (glucose, insulin, amino acids, l-[1-^13^C]-leucine enrichments, l-[1-^13^C]- and l-[ring-^2^H_5_]-phenylalanine enrichments, l-[1-^13^C]-, l-[ring-3,5-^2^H_2_]-, and l-[ring-^2^H_4_]- tyrosine enrichments, and mixed plasma proteins) as well as muscle (mixed muscle protein l-[1-^13^C]-leucine and l-[1-^13^C]- and l-[ring-^2^H_5_]-phenylalanine enrichments) data.

### Calculations

Ingestion of l-[1-^13^C]-phenylalanine-labeled protein, intravenous infusion of l-[ring-^2^H_5_]-phenylalanine and l-[ring-3,5-^2^H_2_]-tyrosine, and blood sample enrichment values were used to assess whole-body amino acid kinetics in non–steady state conditions. Total, exogenous, and endogenous phenylalanine rate of appearance (*R*_a_) and plasma availability of dietary protein–derived phenylalanine that appeared in the systemic circulation as a fraction of the total amount of phenylalanine that was ingested (Phe_plasma_) were calculated using modified Steele's equations (38, 39). For complete details, see the Supplemental Methods.

The primary outcome, fractional synthesis rate (FSR) of mixed muscle protein, was calculated by dividing the increment in enrichment of the product, i.e., protein-bound l-[ring-^2^H_5_]-phenylalanine and l-[1-^13^C]-leucine, by the enrichment of the precursor. The muscle FSR (%/h) was calculated as follows:

(1) $$\begin{eqnarray*}
{\rm{FSR\ }} = \frac{{d{E_p}}}{{{E_{{\rm{precursor}}}}t}}{\rm{\ }} \cdot 100
\end{eqnarray*}$$

Where $d{E_p}$ is the Δ increment of protein-bound l-[ring-^2^H_5_]-phenylalanine and l-[1-^13^C]-leucine during incorporation periods, ${E_{{\rm{precursor}}}}$ is the average (weighed means) plasma l-[ring-^2^H_5_]-phenylalanine and l-[1-^13^C]-leucine enrichment during the period for the determination of amino acid incorporation, and *t* reflects the time interval (h) between sampling time points. Basal muscle protein synthesis rates were assessed using the plasma proteins (*t* = −180 min) and the single biopsy approach.

To account for the differences in the l-[1-^13^C]-phenylalanine enrichments in the lesser mealworm– and milk-derived protein (50.8 and 38.3 MPE, respectively), incorporation of the protein-derived l-[1-^13^C]-phenylalanine into de novo muscle protein was corrected for the differences in the dietary protein l-[1-^13^C]-phenylalanine enrichments. Individual l-[1-^13^C]-phenylalanine enrichment values in the WORM group were divided by 50.8 and multiplied by 38.3, to allow direct comparison between the groups.

### Statistical analysis

All data are expressed as means ± SDs. Data were checked and normality and sphericity were confirmed. Baseline characteristics, peak plasma concentrations, incremental AUC (iAUC), and dietary protein–derived amino acid availability were compared between groups using an independent-samples *t* test. For time-dependent plasma variables, repeated-measures ANOVAs with group (MILK or WORM) as a between-subjects factor and time as a within-subjects factor were used. For FSR variables, repeated-measures ANOVAs with group (MILK or WORM) as a between-subjects factor and leg (exercise compared with rest) and time (basal compared with postprandial) as within-subjects factors were used. In case of significant interactions, separate analyses were performed as appropriate (i.e., within each group or within legs, as well as between groups or between legs for every time period separately). In the case of significant time effects, Bonferroni post hoc analyses were performed to locate the differences for plasma variables. Significance was set at *P* < 0.05. Calculations were performed using SPSS version 25.0 (IBM Corp.).

Sample size was calculated with differences in postprandial muscle FSR as the primary outcome measure. Based on studies comparing muscle protein turnover between groups at rest and after exercise (4, 6), we used a difference between groups in muscle FSR of 20% (within-group SD: 16%). Using a power of 80%, a significance level of 0.05, and a dropout rate of 10%, the final number of participants was calculated as *n* = 12/group.

## Results

### Plasma metabolites

Plasma glucose concentrations increased shortly after protein ingestion, and then decreased (peak values: 5.5 ± 0.4 and 5.6 ± 0.6 mmol/L for MILK and WORM, respectively; Time: *P* < 0.001), with no differences between groups (Time × Group: *P* = 0.945; data not shown). Plasma insulin concentrations increased after protein ingestion in both groups (peak values: 20.9 ± 11.6 and 21.2 ± 9.3 mU/L for MILK and WORM, respectively; Time: *P* < 0.001) and returned back to baseline values within 5 h. No significant differences were observed between groups (Time × Group: *P* = 0.223; data not shown).

**FIGURE 1:**
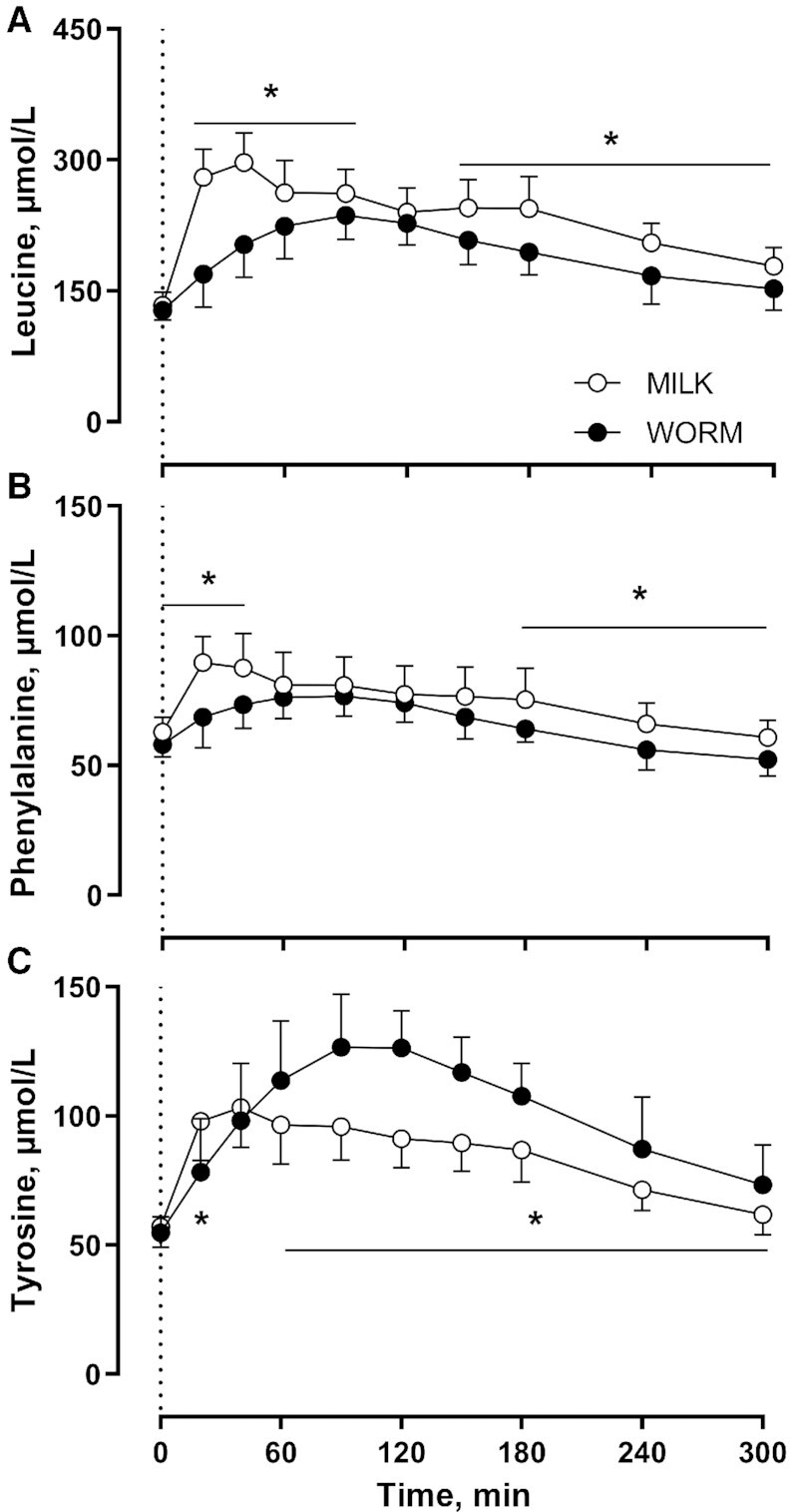
Plasma leucine (A), phenylalanine (B), and tyrosine (C) concentrations after ingestion of milk- (MILK; *n* = 12) or lesser mealworm– (WORM; *n* = 12) derived protein during 5 h of recovery from a single bout of unilateral exercise in healthy, young men. The dotted line represents the time of protein ingestion. Values represent means ± SDs. Data were analyzed using repeated-measures (Time × Group) ANOVA and separate analyses were performed when a significant interaction was detected. Bonferroni post hoc testing was used to detect differences between groups. Time × Group interactions were observed for plasma leucine, phenylalanine, and tyrosine concentrations (all *P* < 0.05). *MILK significantly different from WORM (*P* < 0.05). MILK, 30 g milk protein concentrate; WORM, 30 g lesser mealworm–derived protein.

Plasma leucine (**Figure 1**A), phenylalanine (Figure 1B), and tyrosine (Figure 1C) concentrations increased after MILK and WORM protein ingestion (Time: *P* < 0.001). Plasma leucine concentrations were higher for MILK than for WORM at all time points, except *t* = 0 and 120 min (Time × Group: *P* < 0.001). Peak leucine concentrations were 27% higher for MILK than for WORM (*P* < 0.001). Plasma phenylalanine concentrations were higher for MILK than for WORM, except between *t* = 60 and 150 min (Time × Group: *P* = 0.002). Peak plasma phenylalanine concentrations were 15% higher for MILK than for WORM (*P* = 0.007). Plasma tyrosine concentrations were higher for WORM than for MILK from *t* = 60 through 300 min (Time × Group: *P* < 0.001). Peak plasma tyrosine concentrations were 22% higher for WORM than for MILK (*P* < 0.001).

**FIGURE 2:**
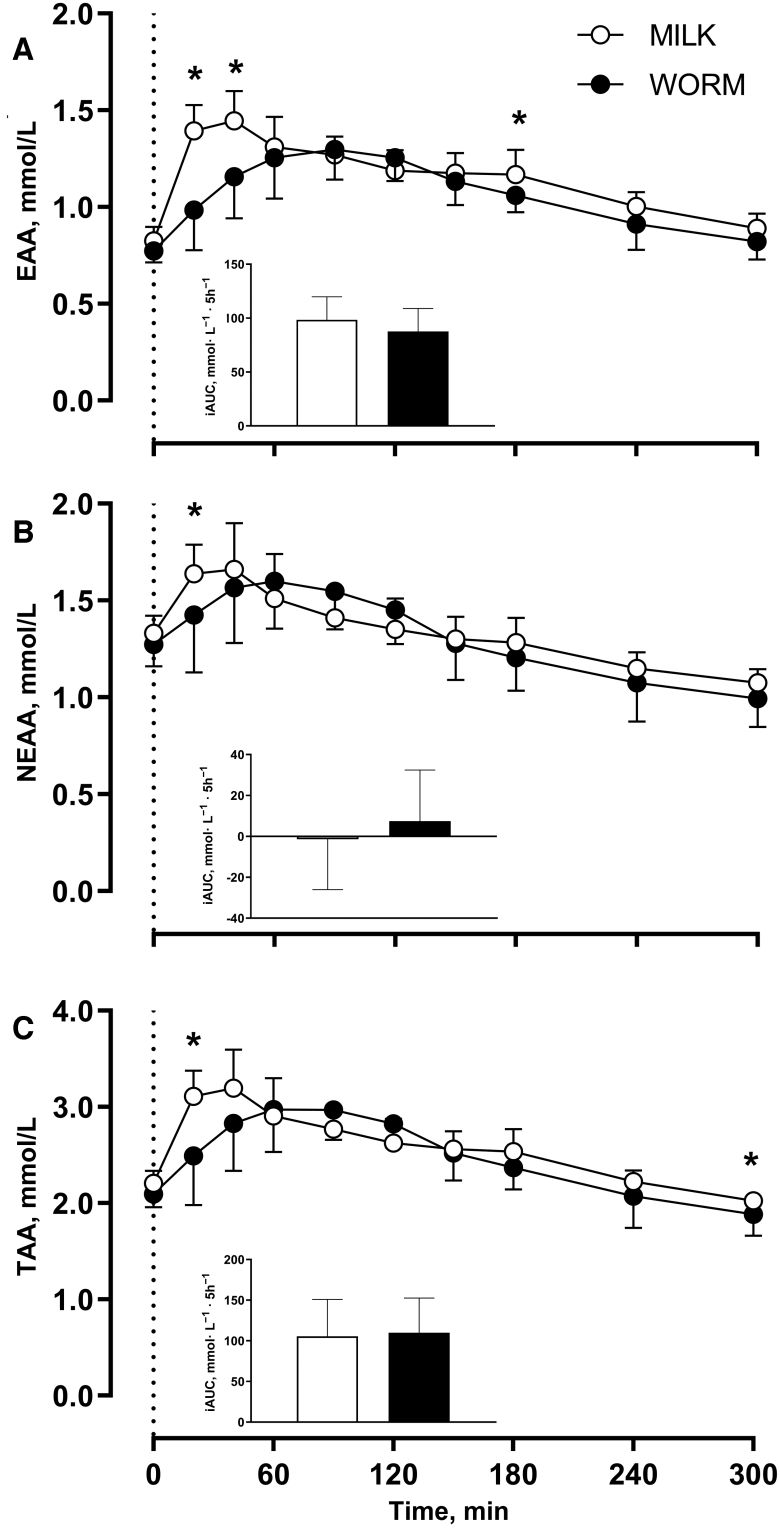
Plasma EAA (A), NEAA (B), and TAA (C) concentrations and iAUCs after ingestion of milk- (MILK; *n* = 12) or lesser mealworm– (WORM; *n* = 12) derived protein during 5 h of recovery from a single bout of unilateral exercise in healthy, young men. The dotted line represents the time of protein ingestion. Values represent means ± SDs. Data were analyzed using repeated-measures (Time × Group) ANOVA and separate analyses were performed when a significant interaction was detected. Bonferroni post hoc testing was used to detect differences between groups. Time × Group interactions were observed for plasma EAAs, NEAAs, and TAAs (all *P* < 0.05). *MILK significantly different from WORM (*P* < 0.05). EAA, essential amino acid; MILK, 30 g milk protein concentrate; NEAA, nonessential amino acid; TAA, total amino acid; WORM, 30 g lesser mealworm–derived protein.

**Figure 2** depicts concentrations of plasma essential amino acids (EAAs; Figure 2A), nonessential amino acids (NEAAs; Figure 2B), and total amino acids (TAAs; Figure 2C). Plasma EAA concentrations increased after protein ingestion (Time: *P* < 0.001) and were higher for MILK than for WORM at *t* = 20, 40, and 180 min (Time × Group: *P* < 0.001). Peak plasma EAA concentrations were 12% higher for MILK than for WORM (*P* = 0.005) with no significant differences for postprandial EAA iAUC between groups (*P* = 0.231). Plasma NEAA concentrations increased after protein ingestion (Time: *P* < 0.001) and were higher for MILK than for WORM at *t* = 20 min (Time × Group: *P* = 0.003). However, the peak plasma NEAA concentrations and the postprandial NEAA iAUC did not differ between groups (*P* = 0.466 and *P* = 0.397, respectively). Plasma TAA concentrations increased after protein ingestion (Time: *P* < 0.001) and were higher for MILK than for WORM at *t* = 20 and 300 min (Time × Group: *P* = 0.001). However, the peak plasma TAA concentrations and the postprandial TAA iAUC did not differ between groups (*P* = 0.181 and *P* = 0.812, respectively).

### Whole-body amino acid kinetics and metabolism

**FIGURE 3:**
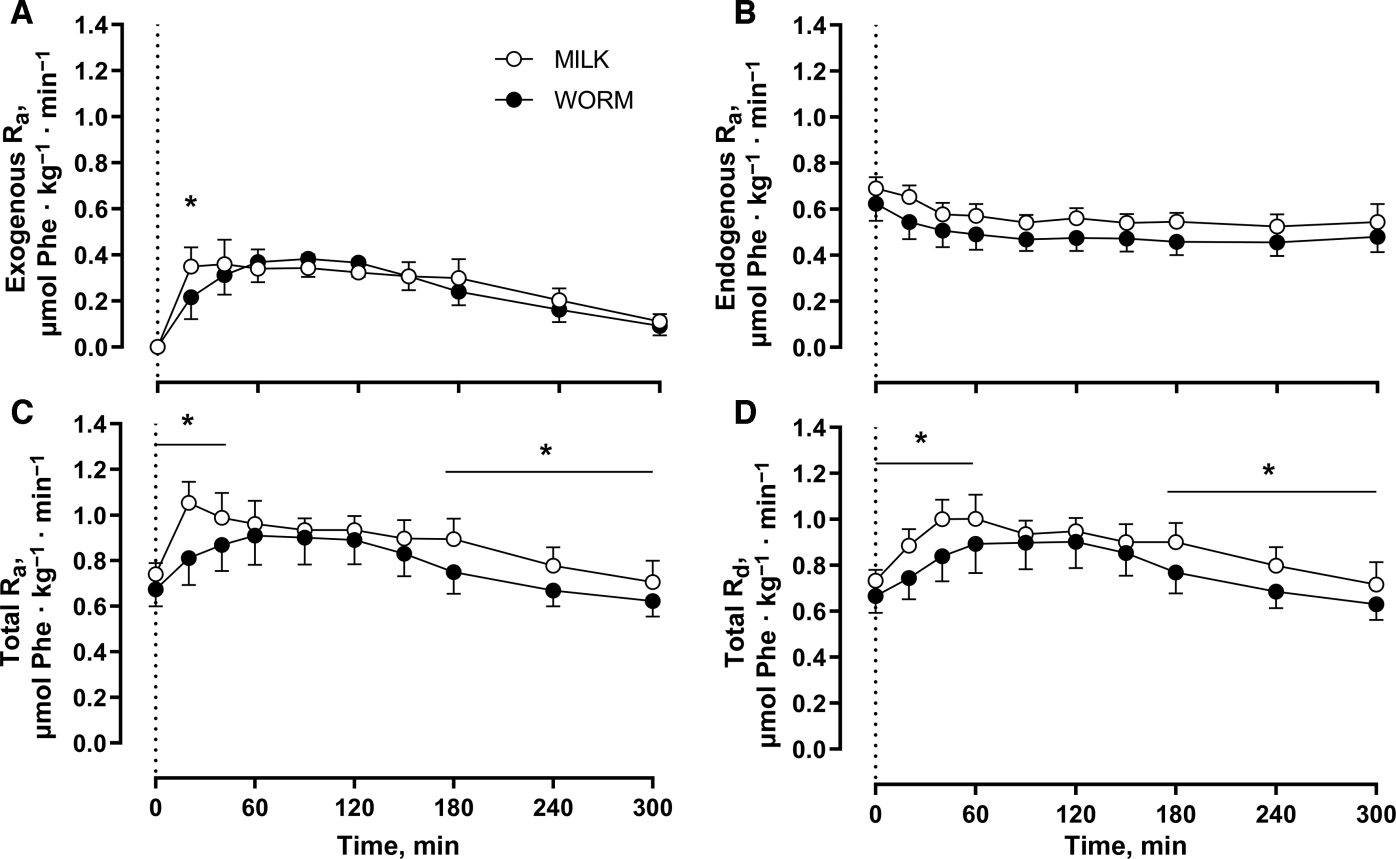
Exogenous phenylalanine *R*_a_ (A), endogenous phenylalanine *R*_a_ (B), total phenylalanine *R*_a_ (C), and total plasma phenylalanine *R*_d_ (D) after ingestion of milk- (MILK; *n* = 12) or lesser mealworm– (WORM; *n* = 12) derived protein during 5 h of recovery from a single bout of unilateral exercise in healthy, young men. The dotted line represents the time of protein ingestion. Values represent means ± SDs. Data were analyzed using repeated-measures (Time × Group) ANOVA and separate analyses were performed when a significant interaction was detected. Bonferroni post hoc testing was used to detect differences between groups. Time × Group interactions were observed for exogenous phenylalanine *R*_a_, total phenylalanine *R*_a_, and total phenylalanine *R*_d_ (all *P* < 0.05). *MILK significantly different from WORM (*P* < 0.05). MILK, 30 g milk protein concentrate; *R*_a_, rate of appearance; *R*_d_, rate of disappearance; WORM, 30 g lesser mealworm–derived protein.

**Figure 3** shows exogenous (Figure 3A), endogenous (Figure 3B), and total plasma phenylalanine *R*_a_ (Figure 3C) and total plasma phenylalanine rates of disappearance (*R*_d_) (Figure 3D). Exogenous phenylalanine *R*_a_ increased after protein ingestion and remained elevated over the entire postprandial period (Time: *P* < 0.001) with the exogenous phenylalanine *R*_a_ being higher for MILK than for WORM at *t* = 20 min (Time × Group: *P* = 0.009). Endogenous phenylalanine *R*_a_ decreased after protein ingestion and remained below baseline values during the entire postprandial period (Time: *P* < 0.001), with no significant differences between groups (Time × Group: *P* = 0.269). After protein ingestion, total phenylalanine *R*_a_ increased and remained elevated for 150 min (Time: *P* < 0.001) and the total phenylalanine *R*_a_ was higher for MILK than for WORM at *t* = 0, 20, 40, and 180–300 min (Time × Group: *P* = 0.001). Total phenylalanine *R*_d_ increased after protein ingestion and remained elevated for 180 min (Time: *P* < 0.001), and was higher for MILK than for WORM at *t* = 0–60 and *t* = 180–300 min (Time × Group: *P* = 0.022).

**FIGURE 4:**
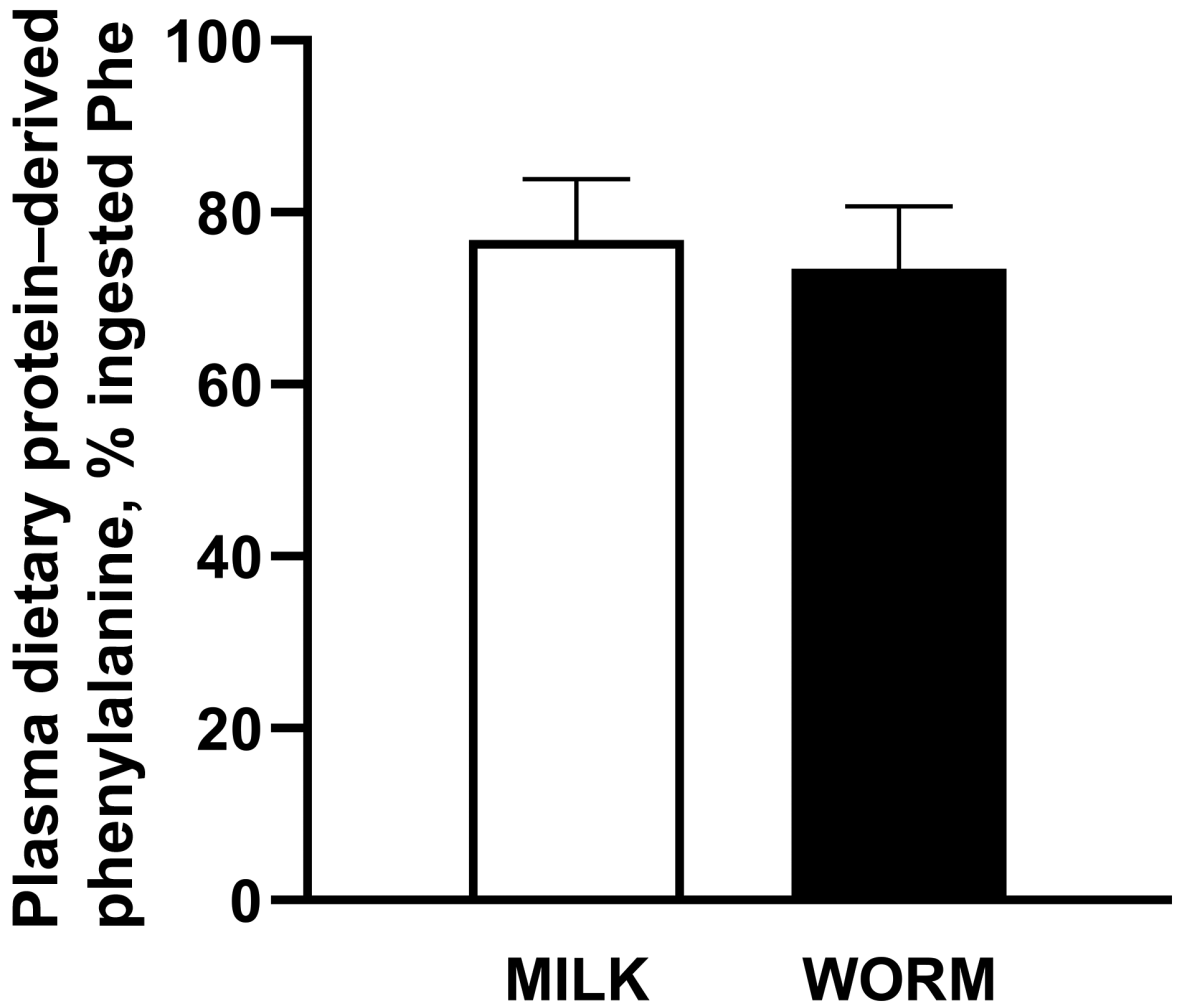
Dietary protein–derived phenylalanine (expressed as a percentage of total amount of protein-bound phenylalanine ingested) released in the circulation during 5 h after ingestion of milk- (MILK; *n* = 12) or lesser mealworm– (WORM; *n* = 12) derived protein in healthy, young men. Values represent means ± SDs. Data were analyzed using an independent *t* test. MILK, 30 g milk protein concentrate; WORM, 30 g lesser mealworm–derived protein.

Plasma dietary protein–derived phenylalanine availability over the 5 h postprandial period (calculated as a fraction of the total amount of ingested phenylalanine) was 73% ± 7% and 77% ± 7% for WORM and MILK, respectively, and did not differ between groups (*P* = 0.263) (**Figure 4**). The calculated absolute amount of dietary protein–derived amino acids that appeared in the circulation during the 5-h postprandial period was 22.0 ± 0.6 and 23.0 ± 0.6 g for WORM and MILK, respectively. Also, when plasma dietary protein–derived amino acid release was expressed as availability over 0–2 h (12.4 ± 2.7 and 12.2 ± 2.1 g) and 2–5 h (9.6 ± 1.8 and 10.8 ± 1.7 g), WORM and MILK were not significantly different (*P* = 0.790 and *P* = 0.084, respectively).

Whole-body protein synthesis rates (0.77 ± 0.06 compared with 0.68 ± 0.08 µmol Phe · kg^−1^ · min^−1^) were 13% higher and breakdown rates (0.55 ± 0.04 compared with 0.48 ± 0.06 µmol Phe · kg^−1^ · min^−1^) were 16% higher for MILK than for WORM (*P* = 0.005 and *P* = 0.002, respectively). However, whole-body protein oxidation rates (0.10 ± 0.02 compared with 0.09 ± 0.05 µmol Phe · kg^−1^ · min^−1^) and net balance (0.21 ± 0.04 compared with 0.20 ± 0.06 µmol Phe · kg^−1^ · min^−1^) did not differ between MILK and WORM (*P* = 0.782 and *P* = 0.426, respectively).

### Mixed muscle protein synthesis rates

**FIGURE 5:**
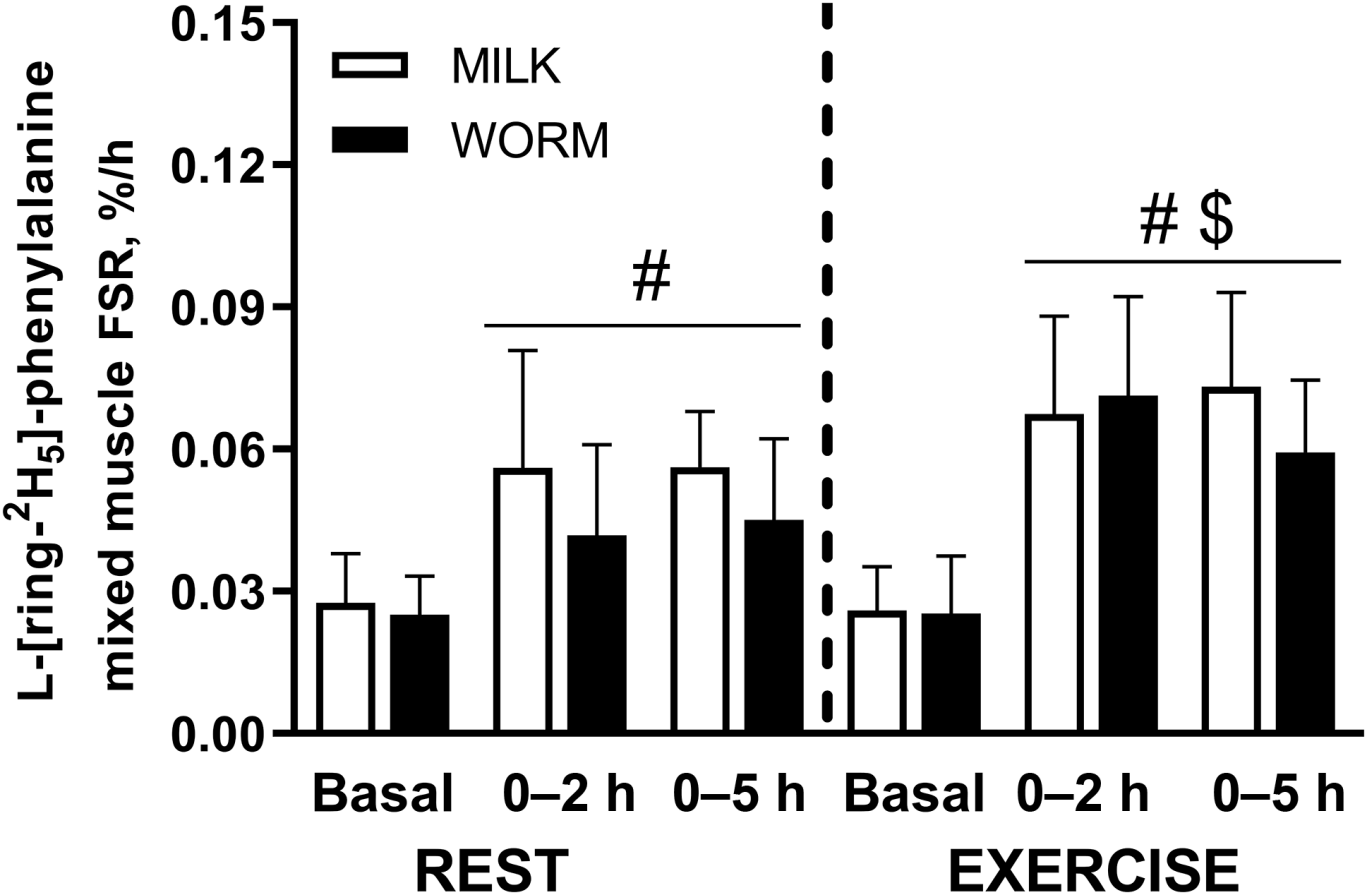
Mixed muscle protein FSRs assessed using l-[ring-^2^H_5_]-phenylalanine after ingestion of milk- (MILK; *n* = 12) or lesser mealworm– (WORM; *n* = 11) derived protein for both REST and EXERCISE in healthy, young men. Values represent means ± SDs. Data were analyzed using repeated-measures (Time × Leg × Group) ANOVA, and separate analyses were performed when a significant interaction was detected. A Time × Leg × Group interaction was absent (*P* > 0.05). A Time × Leg interaction was observed (*P* < 0.05). ^#^Both 0–2 h and 0–5 h postprandial FSR significantly different from basal (*P* < 0.05); ^$^postprandial FSR in EXERCISE significantly different from REST (*P* < 0.05). EXERCISE, exercised leg; FSR, fractional synthesis rate; MILK, 30 g milk protein concentrate; REST, rested leg; WORM, 30 g lesser mealworm–derived protein.

**Figure 5** shows mixed muscle protein synthesis rates calculated based upon l-[ring-^2^H_5_]-phenylalanine. No Time × Leg × Group interactions were observed (*P* = 0.114 and *P =* 0.549 for 0–2 h and 0–5 h, respectively). A significant Leg × Time interaction showed that the increase in muscle protein synthesis rates from basal to postprandial was greater in the EXERCISE than in the REST leg, for both 0–2 h and 0–5 h (both *P* < 0.001).

In the REST leg, muscle protein synthesis rates based on l-[ring-^2^H_5_]-phenylalanine (Figure 5) increased from basal to postprandial (0–2 h) after both WORM (from 0.025 ± 0.008%/h to 0.042 ± 0.019%/h) and MILK (from 0.028 ± 0.010%/h to 0.056 ± 0.025%/h) protein ingestion (Time: *P* < 0.001), with no significant differences between groups (Time × Group: *P* = 0.218). In the EXERCISE leg, postexercise muscle protein synthesis rates based on l-[ring-^2^H_5_]-phenylalanine (Figure 5) increased from basal to postprandial (0–2 h) after both WORM (from 0.025 ± 0.012%/h to 0.071 ± 0.021%/h) and MILK (from 0.026 ± 0.009%/h to 0.067 ± 0.021%/h) protein ingestion (Time: *P* < 0.001), with no significant differences between groups (Time × Group: *P* = 0.588). Similarly, muscle protein synthesis rates based on l-[ring-^2^H_5_]-phenylalanine increased from basal to postprandial (0–5 h) after both WORM (0.045 ± 0.017%/h) and MILK (0.056 ± 0.012%/h) protein ingestion (Time: *P* < 0.001) at REST, as well as after EXERCISE (Time: *P* < 0.001; WORM: 0.059 ± 0.015%/h and MILK: 0.073 ± 0.020%/h), with no significant differences between groups (Time × Group: *P* = 0.089 and *P* = 0.088, respectively).

In line with the data for l-[ring-^2^H_5_]-phenylalanine, no Time × Leg × Group interactions were observed for postprandial muscle protein synthesis rates based on l-[1-^13^C]-leucine (*P* = 0.372 and *P* = 0.684 for 0–2 h and 0–5 h, respectively), but significant Leg × Time interactions showed that the increase in muscle protein synthesis based on l-[1-^13^C]-leucine from basal to postprandial was greater in the EXERCISE than in the REST leg, for both 0–2 h (*P* = 0.022) and 0–5 h (*P* < 0.001). In the early postprandial period (0–2 h), muscle protein synthesis rates based on l-[1-^13^C]-leucine increased to 0.030 ± 0.026%/h and 0.048 ± 0.028%/h after WORM and MILK protein ingestion at REST (Time: *P* < 0.001), respectively, and to 0.060 ± 0.031%/h and 0.050 ± 0.041%/h after EXERCISE (Time: *P* < 0.001), respectively, with no significant differences between groups (Time × Group: *P* = 0.433 and *P* = 0.871, respectively). Similarly, muscle protein synthesis rates based on l-[1-^13^C]-leucine increased from basal to postprandial (0–5 h) after both WORM (0.042 ± 0.020%/h) and MILK (0.055 ± 0.017%/h) protein ingestion (Time: *P* < 0.001) at REST, as well as after EXERCISE (Time: *P* < 0.001; WORM: 0.061 ± 0.013%/h and MILK: 0.073 ± 0.017%/h), with no significant differences between groups (Time × Group: *P* = 0.552 and *P* = 0.394, respectively).

**FIGURE 6:**
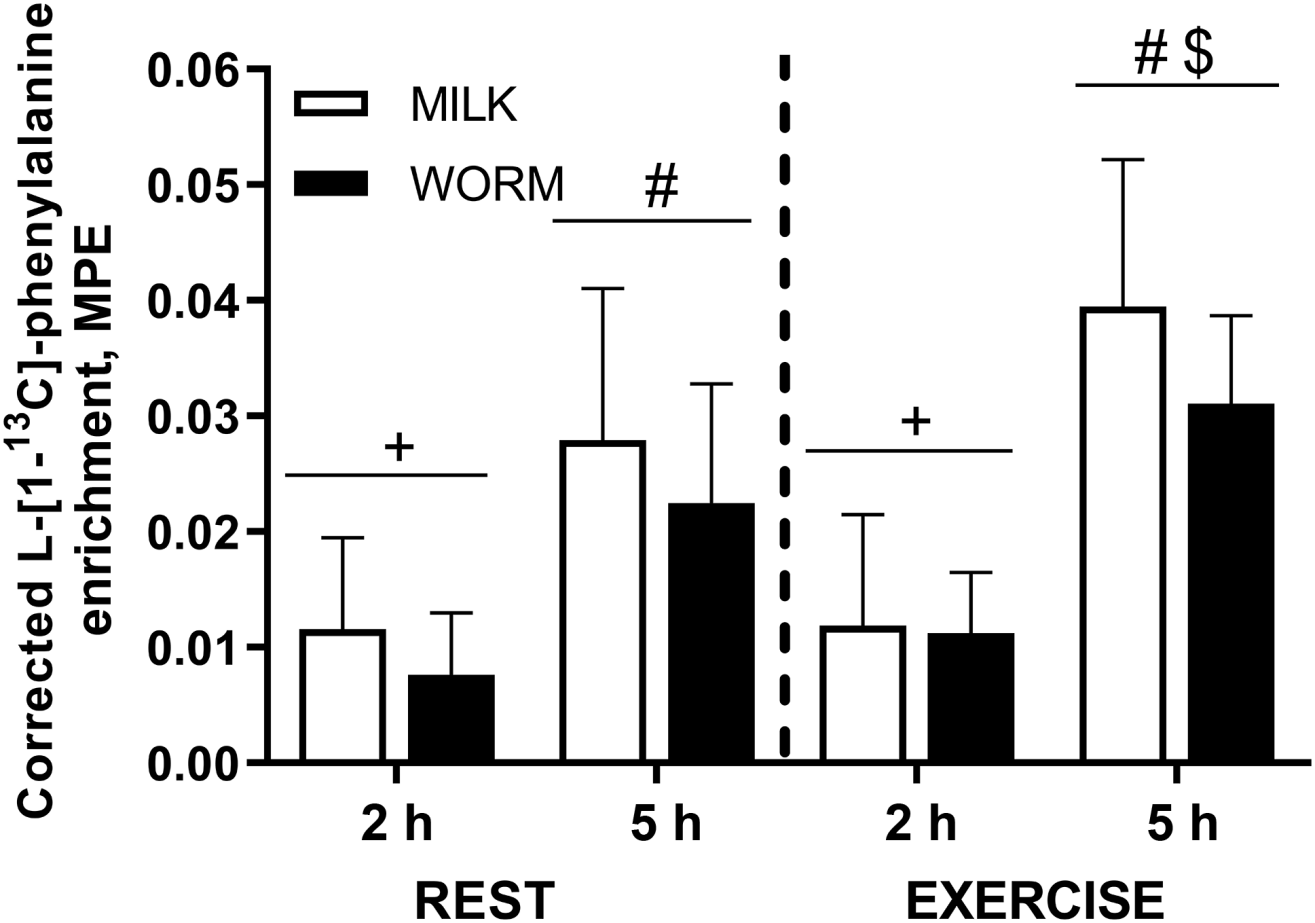
Corrected l-[1-^13^C]-phenylalanine enrichment in mixed muscle protein over time after ingestion of milk- (MILK; *n* = 12) or lesser mealworm– (WORM; *n* = 11) derived protein for both REST and EXERCISE in healthy, young men. Values for the WORM group were multiplied by 0.75 to correct for the difference in enrichment in the MILK (38.3 MPE) and WORM (50.8 MPE) protein. Values represent means ± SDs. Data were analyzed using repeated-measures (Time × Leg × Group) ANOVA, and separate analyses were performed when a significant interaction was detected. A Time × Leg × Group interaction was absent (*P* > 0.05). A Time × Leg interaction was observed (*P* < 0.05). ^+^*t* = 2 h significantly different from *t* = 0 h (*P* < 0.05); ^#^*t* = 5 h significantly different from *t* = 0 and *t* = 2 h (*P* < 0.05); ^$^REST significantly different from EXERCISE for that time point (*P* < 0.05). EXERCISE, exercised leg; MILK, 30 g milk protein concentrate; MPE, mole percentage excess; REST, rested leg; WORM, 30 g lesser mealworm–derived protein.

Incorporation of protein-derived l-[1-^13^C]-phenylalanine into de novo muscle protein during the postprandial period increased after protein ingestion at REST (Time: *P* < 0.001) and after EXERCISE (Time: *P* < 0.001), but did not differ between groups (Time × Group: *P* = 0.623 and Time × Group: *P* = 0.692, respectively; data not shown). To account for differences in the l-[1-^13^C]-phenylalanine enrichments in the WORM and MILK protein (50.8 compared with 38.3 MPE, respectively), incorporation of protein-derived l-[1-^13^C]-phenylalanine into de novo muscle protein was corrected for the differences in dietary protein l-[1-^13^C]-phenylalanine enrichments. **Figure 6** shows the relative incorporation of dietary protein–derived l-[1-^13^C]-phenylalanine into de novo muscle protein. Relative muscle protein-bound l-[1-^13^C]-phenylalanine incorporation increased after protein ingestion at REST (Time: *P* < 0.001) and during recovery from EXERCISE (Time: *P* < 0.001), with no significant differences between groups (Time × Group: *P* = 0.302 and Time × Group: *P* = 0.110, respectively). At *t* = 5 h relative muscle protein-bound l-[1-^13^C]-phenylalanine enrichments were higher for the EXERCISE than for the REST leg (*P* < 0.001).

## Discussion

This study shows that ingestion of lesser mealworm and milk protein concentrate are both followed by rapid protein digestion and amino acid absorption, with >70% of the ingested protein-bound phenylalanine being released into the circulation during the 5-h postprandial period. Ingestion of both mealworm and milk protein concentrate increased muscle protein synthesis rates both at rest and during recovery from resistance-type exercise, with no differences between protein sources.

Conventional animal-based protein sources such as meat, eggs, and milk are considered high-quality protein sources because they are highly digestible and meet all of the current EAA requirements (40). However, production of sufficient amounts of these animal-based protein sources is not feasible to meet the growing global protein demands (41). Consequently, there is an increasing interest in the production of alternative and more sustainable dietary protein sources. Edible insects have been identified as a protein-dense food source that may be produced more sustainably at a viable commercial scale for human consumption (19, 21). From the wide array of edible insects, lesser mealworms (*A. diaperinus*) have been reported to provide a source of high-quality protein containing high amounts of EAAs (18–21). In agreement with previous literature (18, 20, 42), we show that lesser mealworm protein has a relatively high EAA content and a leucine content comparable with that of milk protein (Table 2). This implies that lesser mealworm may also have anabolic properties that are not distinct from high-quality animal-derived protein sources.

In addition to amino acid composition, protein digestion and amino acid absorption kinetics represent another important factor that determines the capacity of a protein source to stimulate postprandial muscle protein synthesis (3, 6–8). After lesser mealworm–derived protein ingestion, we observed a rapid rise in circulating plasma amino acid concentrations that remained elevated over the subsequent hours (Figures 1, 2), which is in line with previous data (42). Because postprandial plasma amino acid responses do not necessarily reflect protein digestion and amino acid absorption, we combined the ingestion of specifically produced intrinsically l-[1-^13^C]-phenylalanine labeled protein with continuous intravenous l-[ring-^2^H_5_]-phenylalanine infusion. Upon ingestion of the lesser mealworm– and milk-derived protein we observed a rapid rise in the release of exogenous protein–derived phenylalanine into the circulation (Figure 3). The postprandial rise in release of exogenous protein–derived phenylalanine was greater after milk than after lesser mealworm–derived protein during the early postprandial phases. Throughout the full 5-h postprandial period 73% ± 7% and 77% ± 7% of the ingested protein-bound phenylalanine had been released into the circulation after the ingestion of 30 g lesser mealworm– and milk-derived protein, respectively (*P* > 0.05) (Figure 4). These data are the first that we know of to show that insect-derived protein can be rapidly digested and effectively absorbed, with a postprandial rise in protein-derived amino acid availability in the circulation that is not different from a high-quality dairy protein source.

It has been well established that protein ingestion stimulates muscle protein synthesis (43, 44), with the postprandial rise in circulating EAAs (45, 46) and leucine in particular (47, 48) being responsible for activating the muscle protein synthetic machinery. In this study, we measured the incorporation of l-[ring-^2^H_5_]-phenylalanine and l-[1-^13^C]-leucine in muscle protein to assess postabsorptive and postprandial muscle tissue protein FSRs. Using the l-[ring-^2^H_5_]-phenylalanine tracer, we observed a substantial increase in muscle protein synthesis rates after both mealworm and milk protein concentrate ingestion (Figure 5). Exercise before protein intake sensitizes skeletal muscle tissue to the anabolic properties of amino acids, resulting in an even greater postprandial increase in muscle protein synthesis rates (11, 49). In the present study, participants performed a single bout of 1-legged resistance-type exercise before protein ingestion, allowing us to assess muscle protein synthesis rates both at rest and during recovery from exercise. In agreement, exercise stimulated muscle protein synthesis rates resulting in an even greater increase in postprandial muscle protein synthesis rates after ingestion of both protein sources (Figure 5). No differences were observed between treatments, implying that the lesser mealworms represent a readily accessible protein source with strong anabolic properties that do not seem to differ from a high-quality protein source like milk.

In the current study, participants ingested specifically produced, intrinsically labeled lesser mealworm– and milk-derived protein with very high l-[1-^13^C]-phenylalanine enrichments (50.8 and 38.3 MPE, respectively). We observed no significant differences in the relative incorporation of dietary protein–derived l-[1-^13^C]-phenylalanine into skeletal muscle protein (Figure 6). Of course, prior exercise further increased the uptake of dietary protein–derived l-[1-^13^C]-phenylalanine into muscle protein, confirming the impact of muscle contraction on stimulating amino acid uptake and subsequent de novo incorporation into muscle protein. These data demonstrate that both the mealworm and the milk protein concentrate provide us with required amino acids as precursors for de novo muscle protein synthesis, confirming that we are what we just ate (26), whether that is milk or mealworm.

In the present study we provided subjects with a meal-sized serving of milk- or lesser mealworm–derived protein. Previous work has reported that ingestion of 20 g high-quality protein is sufficient to maximize postprandial muscle protein synthesis rates in healthy, young adults (4, 50). Therefore, it could be speculated that ingestion of smaller doses of lesser mealworm–derived protein could have revealed (greater) differences in the muscle protein synthetic response to milk- compared with lesser mealworm–derived protein ingestion. However, in the present study we were able to show similar efficacy of both protein digestion and amino acid absorption (Figure 4) and we were unable to detect any measurable differences in postprandial muscle protein synthesis rates (Figure 5). These observations differ from previous work comparing dairy with plant-based protein isolates, where it was shown that substantially more wheat protein has to be ingested to allow a muscle protein synthetic response equivalent to that seen after the ingestion of 35 g dairy protein (15). Clearly, the high quality of the insect-derived proteins (18) compared with existing commercial plant-based protein concentrates and isolates (51) will be of particular relevance in populations where protein quality is of great(er) importance. The latter includes people that consume less protein and suffer from anabolic resistance (52), such as the older and/or more clinically compromised populations. A more sustainable protein source without the obvious limitations of low digestibility, low EAA content, and/or specific amino acid deficiency would prove to be highly attractive. Together with the additional benefits of insects containing high amounts of micronutrients (53) and antioxidants (54), our data provide a strong incentive for incorporating insects into the Western diet. The consumption of insects is already common for >2 billion people worldwide, mainly in Asia, Africa, and South America (53). It will be interesting to see insects and insect-derived proteins becoming a major protein source in our future food supply (55).

In conclusion, ingestion of a meal-like amount of lesser mealworm is followed by rapid protein digestion and amino acid absorption and a substantial increase in muscle protein synthesis rates both at rest and during recovery from exercise. The postprandial protein handling of lesser mealworm–derived protein does not differ compared with ingestion of the same amount of milk protein in vivo in humans, which shows that insects can provide a viable, high-quality protein source for human consumption.

## Supplementary Material

nqab115_Supplemental_File

## Data Availability

Data described in the article, code book, and analytic code will be made available upon request pending application and approval.

## Notes

Supported by an in-kind contribution in the form of stable isotope tracers from Cambridge Isotope Laboratories for the production of intrinsically labeled lesser mealworm.

JJAvL is on the Board of Advisors for Protifarm. Protifarm produces large quantities of sustainable, high-quality insect ingredients for the food and pharmaceutical industry and is based in Ermelo, Netherlands.

Supplemental Methods are available from the “Supplementary data” link in the online posting of the article and from the same link in the online table of contents at https://academic.oup.com/ajcn/.

Abbreviations used: EAA, essential amino acid; En%, energy percent; FSR, fractional synthesis rate; iAUC, incremental AUC; MILK, 30 g milk protein concentrate; MPE, mole percentage excess; NEAA, nonessential amino acid; *R*_a_, rate of appearance; *R*_d_, rate of disappearance; TAA, total amino acid; WORM, 30 g lesser mealworm–derived protein; 1-RM, 1 repetition maximum.
