# Supplementary material for: Insects are a viable protein source for human consumption: from insect protein digestion to postprandial muscle protein synthesis in vivo in humans: a double-blind randomized trial
Source: Am J Clin Nutr. 2021 May 21;114(3):934–44. doi: 10.1093/ajcn/nqab115 (PMC8408844; doi:10.1093/ajcn/nqab115)
Supplement: nqab115_Supplemental_File [file nqab115_supplemental_file.docx]

This is the online supplementary material to the manuscript: *Insects are a viable protein source for human consumption: from insect protein digestion to post-prandial muscle protein synthesis in vivo in humans: a double-blind randomized trial* by Wesley J.H. Hermans, Joan M. Senden, Tyler A. Churchward-Venne, Kevin J.M. Paulussen, Cas J. Fuchs, Joey S.J. Smeets, Joop J.A. van Loon, Lex B. Verdijk and Luc J.C. van Loon

*List of abbreviations*

| GC-C-IRMS | Gas chromatography-combustion-isotope ratio mass spectrometry |
| --- | --- |
| GC-P-IRMS | Gas chromatography-pyrolysis-isotope ratio mass spectrometry |
| GC-MS | Gas chromatography-mass spectrometry |
| MTBSTFA | *N-tert-*butyldimethylsilyl*-N-*methyltrifluoroacetamide |
| PCA | Perchloric acid |
| Ra | Rate of appearance |
| Rd | Rate of disappearance |
| SD | Standard deviation |
| TBDMS | Tert-butyldimethylsilyl |
| UPLC-MS | Ultra-performance liquid chromatograph mass spectrometry |

*Plasma analyses*

Plasma glucose and insulin concentrations were measured using commercially available kits (ref. no. A11A01667, Glucose HK CP, ABX Diagnostics, Montpellier, France; and ref. no. HI-14K, Millipore, St. Louis, MO, respectively). Plasma amino acid concentrations were quantified using UPLC-MS (ACQUITY UPLC H-Class with QDa; Waters, Saint-Quentin, France). A total of 50 µL of blood plasma was deproteinized using 100 µL of 10% SSA with 50 µM of MSK-A2 internal standard (Cambridge Isotope Laboratories, Massachusetts, USA). Subsequently, 50 µL of ultra-pure demineralized water was added and samples were centrifuged (15 min at 21000*g*). After centrifugation, 10 µL of supernatant was added to 70 µL of Borate reaction buffer (Waters, Saint-Quentin, France). In addition, 20 µL of AccQ-Tag derivatizing reagent solution (Waters, Saint-Quentin, France) was added after which the solution was heated to 55°C for 10 min. An aliquot of 1 µL was injected and measured using UPLC-MS.

Plasma leucine, phenylalanine, and tyrosine concentrations and enrichments were determined by GC-MS (Agilent 7890A GC/5975C; MSD, Wilmington, DE, USA). Specifically, internal standards of [U-^13^C_6_]-leucine, [U-^13^C_9_^15^N]-phenylalanine, and [U-^13^C_9_^15^N]-tyrosine were added to the plasma samples. Plasma samples were deproteinized with dry 5-sulphosalicylic acid. Free amino acids were purified using cation exchange resin (Dowex AG 50W-X8 resin, mesh size: 100–200 µm, ionic form: hydrogen; Bio-Rad Laboratories, Hercules, CA, USA). The purified amino acids were converted into tert-butyldimethylsilyl (TBDMS) derivatives with *N-tert*-butyldimethylsilyl-*N*-methyltrifluoroacetamide (MTBSTFA) before analysis by GC-MS. The plasma leucine, phenylalanine, and tyrosine concentrations were determined using selective ion monitoring at mass/charge (m/z) 302 and 308 for unlabeled and [U-^13^C_6_] labeled-leucine, 336 and 346 for unlabeled and [U-^13^C_9_^15^N] labeled phenylalanine, and 466 and 476 for unlabeled and [U-^13^C_9_^15^N]-tyrosine] labeled tyrosine, respectively. The plasma leucine, phenylalanine and tyrosine ^13^C and ^2^H enrichments were determined at m/z 302 and 303 for unlabeled and labeled ([1-^13^C]-leucine), respectively, at m/z 336, 337, and 341 for unlabeled and labeled ([1-^13^C]- and [ring-^2^H_5_]) phenylalanine, and at m/z 466, 467, 468, and 470 for unlabeled and labeled ([1-^13^C]-, [ring-^2^H_2_]- and [ring-^2^H_4_]-) tyrosine, respectively. Standard regression curves were applied from a series of known standard enrichment values against the measured values to assess the linearity of the mass spectrometer and to account for any isotope fractionation.

Plasma protein was extracted from basal blood samples by adding 20% perchloric acid (PCA) to a final concentration of 2%. Samples were centrifuged at 1000*g* at 4°C for 20 min after which the supernatant was removed. The mixed plasma protein pellet was washed with 3 washes of 2% PCA, after which the supernatant was removed. The protein pellet was hydrolyzed after adding 6 M HCl by heating at 120°C for 15-18 h. The hydrolysed protein fraction was then dried under a nitrogen stream while being heated to 120°C. Thereafter, the hydrolyzed mixed plasma protein samples were processed via the same procedures as the muscle tissue analyses.

*Muscle tissue analyses*

For measurement of L-[1-^13^C]-leucine, L-[1-^13^C]-phenylalanine and L-[ring-^2^H_5_]-phenylalanine enrichments in the muscle protein-bound amino acid pool, 50-70 mg wet muscle tissue was freeze-dried. Collagen, blood, and other non-muscle fiber materials were removed from the muscle fibers under a light microscope. The isolated muscle fiber mass was weighed and 35 volumes (7x wet weight of isolated muscle fibers x wet-to-dry ratio 5:1) of ice cold 2% PCA were added. Thereafter, the tissue was sonicated and centrifuged. The protein pellet was washed with 3 additional 1.5 mL washes of 2% PCA, hydrolysed with 6M HCl at 120°C for 15-18 h, and then dried under a nitrogen steam while heated to 120°C. Next, 50% acetic acid solution was added, and the hydrolyzed protein was passed over a cation exchange resin (Dowex AG 50W-X8, 100–200 mesh hydrogen form: Bio-Rad, Hercules, CA) using 2M NH_4_OH. The eluate was dried, and the purified amino acids were derivatized to their N(O,S)-ethoxycarbonyl ethyl esters. For measurement of L-[1-^13^C]-leucine and L-[1-^13^C]-phenylalanine, derivatized samples were measured using a GC-C-IRMS (MAT 253; Thermo Fisher Scientific, Bremen, Germany) equipped with DB5MS 30m column (No. 122–4762; Agilent) and GC-Isolink monitoring of ion masses 44, 45, and 46. For measurement of L-[ring-^2^H_5_]-phenylalanine, derivatized samples were measured using a GC-P-IRMS (MAT 253; Thermo Fisher Scientific, Bremen, Germany) equipped with a DB17MS 60m column with 5m pre-column (No. 122–4762; Agilent) and GC-Isolink, monitoring of ion masses 2 and 3. Standard regression curves were applied from a series of known standard enrichment values against the measured values to assess the linearity of the mass spectrometer and to account for any isotope fractionation which may have occurred during the analysis.

*Calculations*

Ingestion of L-[1-^13^C]-phenylalanine-labeled protein, intravenous infusion of L-[ring-^2^H_5_]-phenylalanine and L-[ring-3,5-^2^H_2_]-tyrosine, and blood sample enrichment values were used to assess whole-body amino acid kinetics in non-steady state conditions. Total, exogenous, and endogenous phenylalanine rate of appearance (*R_a_*) and plasma availability of dietary protein-derived phenylalanine that appeared in the systemic circulation as a fraction of total amount of phenylalanine that was ingested (Phe_plasma_) were calculated using modified Steele’s equations (1, 2). These parameters were calculated as follows:

$\mathrm{Total}Ra = \frac{F_{iv}-\left[ pV\cdot C\left( t \right)\cdot\frac{dE_{iv}}{dt} \right]}{E_{iv}\left( t \right)}$ (1)

$\mathrm{Exo}R_{a}=\frac{\mathrm{Total}R_{a} \cdot E_{po}\left( t \right) +\left[ pV \cdot C\left( t \right) \cdot\frac{dE_{po}}{dt} \right]}{E_{prot}}$ (2)

${\mathrm{Endo}R}_{a}= \mathrm{Total}R_{a} - ExoR_{a} - F_{iv}$ (3)

$Phe plasma=\left( \frac{\mathrm{AUC}_{Exo Ra}}{\mathrm{Phe}_{\mathrm{prot}}} \right)\cdot100$ $Pheplasma=\left( \frac{\mathrm{AUC}_{\mathrm{ExoRa}}}{\mathrm{Phe}_{\mathrm{prot}}} \right)\cdot100$ (4)

where *F_iv_* is the intravenous tracer infusion rate (µmol∙kg^-1^∙min^-1^), *pV* (0.125 L∙kg^-1^) is the distribution volume for phenylalanine (1). *C(t)* is the mean plasma phenylalanine concentration between two consecutive time points. *dE_iv_/dt* represents the time-dependent variations of plasma phenylalanine enrichment derived from the intravenous tracer and *E_iv_(t)* is the mean plasma phenylalanine enrichment from the intravenous tracer between two consecutive time points. Exo*R*_a_ represents the plasma entry rate of dietary protein derived phenylalanine, *E*_Po_*(t)* is the mean plasma phenylalanine enrichment for the ingested tracer, *dE_Po_/dt* represents the time-dependent variations of plasma phenylalanine enrichment derived from the oral tracer and *E_Prot_* is the L-[1-^13^C]-phenylalanine enrichment in the dietary protein. Phe_Plasma_ is the percentage of ingested dietary protein derived phenylalanine that becomes available in the plasma and is calculated using Phe_Prot_ and AUC_Exo_*_R_*_a_. Phe_Prot_ is the amount of dietary protein derived phenylalanine ingested and AUC_Exo_*_R_*_a_ represents the area under the curve (AUC) of Exo*R*_a_, which corresponds to the amount of dietary protein derived phenylalanine that appeared in the blood over a 5 h period following ingestion.

Total rate of disappearance of phenylalanine equals the rate of phenylalanine hydroxylation (first step in phenylalanine oxidation) and utilization for protein synthesis. This parameter is calculated as follows:

$R_{d}=Total R_{a}-pV\cdot\frac{\mathrm{dC}}{\mathrm{dt}}$ $\mathrm{TotalR}_{d}=TotalR_{a}-pV\cdot\frac{\mathrm{dC}}{\mathrm{dt}}$
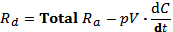
 (5)

Because whole-body *R*_d_ comprises the rate of phenylalanine disappearance from the free amino acid pool in the blood due to protein synthesis and oxidation, whole-body protein synthesis can be calculated as *R*_d_ minus oxidation. Whole-body phenylalanine oxidation can be determined from the conversion (hydroxylation) of L-[ring-^2^H_5_]-phenylalanine to L-[ring-^2^H_4_]-tyrosine. The rate of hydroxylation was calculated by using the following formula:

$\mathrm{Phe}_{\mathrm{hydroxylation}}={\mathrm{Tyr}R}_{a}\cdot\frac{E_{t}\left( t \right)}{E_{p}\left( t \right)}\cdot\frac{{\mathrm{Phe}R}_{d}}{\left( F_{\mathrm{ivp}} + {\mathrm{Phe}R}_{d} \right)}$ (6)

Where Tyr*R_a_* is the total rate of tyrosine appearance based on the rate of appearance of L-[ring-3,5-^2^H_2_]-tyrosine infusion and plasma enrichments. *E*_t_(*t*) and *E*_P_(*t*) are the mean L-[ring-^2^H_4_]-tyrosine and L-[ring-^2^H_5_]-phenylalanine enrichments in plasma between two consecutive time points, respectively, and *F*_ivp_ is the infusion rate of L-[ring-^2^H_5_]-phenylalanine. Whole-body protein synthesis was calculated using:

$Protein Synthesis=R_{d}-\mathrm{Phe}_{\mathrm{hydroxylation}}$ (7)

Whole-body protein net balance was calculated as AUC over the 5 h post-prandial phase using whole-body protein synthesis minus endogenous *R*_a_.

$\mathrm{Phe}_{net balance}=Protein synthesis-\mathrm{Endo}_{Ra}$ (8)

1. Boirie Y, Gachon P, Corny S, Fauquant J, Maubois JL, Beaufrere B. Acute postprandial changes in leucine metabolism as assessed with an intrinsically labeled milk protein. Am J Physiol. 1996;271(6 Pt 1):E1083-91.

2. Dangin M, Guillet C, Garcia‐Rodenas C, Gachon P, Bouteloup‐Demange C, Reiffers‐Magnani K, et al. The rate of protein digestion affects protein gain differently during aging in humans. The Journal of physiology. 2003;549(2):635-44.
